# Different characteristics of infants diagnosed with congenital choledochal malformation prenatally or postnatally

**DOI:** 10.1038/s41598-020-79569-4

**Published:** 2021-01-08

**Authors:** Wei Chen, Jia Geng, Ya-lan Tan, Lian Zhao, Hui-hui Jia, Wan-liang Guo

**Affiliations:** 1 Department of Radiology, Children’s Hospital of Soochow University, Suzhou, 215025 China; 2 Clinical Laboratory, 3rd Hospital of Yulin City, Yulin, 719000 China; 3 Department of Radiology, Cancer Hospital of the University of Chinese Academy of Sciences, Hangzhou, China

**Keywords:** Bile duct disease, Gastrointestinal diseases, Biliary tract disease, Bile duct disease

## Abstract

The general condition, clinical and pathological characteristics, and treatment regimens of patients prenatally and postnatally diagnosed with congenital choledochal malformation (CM) were analyzed in order to investigate the clinical significance of early diagnosis, treatment, and intervention in CM. We retrospectively analyzed 33 children who were admitted to the Children’s Hospital of Soochow University between 1 March 2010 and 31 May 2019, and their diagnosis of CM was confirmed by radiological, surgical and pathological findings. All the patients were under 36 months of age. The patients were divided into prenatally diagnosed and postnatally diagnosed groups. There were 16 and 17 CM patients in the prenatally and postnatally diagnosed groups, respectively, with a preponderance of females in both groups. Compared with the prenatally diagnosed group, the postnatally diagnosed group had a higher incidence of abdominal pain and vomiting (p < 0.05) and higher AST, GGT, and TB levels (p < 0.05). Although postoperative histopathological examination showed inflammation in both groups, congestion in the cyst walls and fibrous tissue hyperplasia were more significant in the postnatally diagnosed group (p < 0.05). In addition, operation time, length of time required to resume a normal diet after surgery, and total length of hospitalization differed between the 2 groups (p < 0.05), with the prenatally diagnosed group having a relatively longer operation time and taking longer to resume a normal diet after surgery. However, the total length of hospitalization in the prenatally diagnosed group was shorter than that in the postnatally diagnosed group. Compared with prenatally diagnosed CM patients, more symptoms, greater severity of symptoms, and more time to recovery after surgery were observed in postnatally diagnosed CM patients.

## Introduction

Congenital choledochal malformation is also known as congenital choledochal cystic dilation, and its clinical presentation varies and most often consists of nonspecific jaundice, abdominal pain, abdominal mass, fever, and vomiting. As the disease progresses, CM can be associated with severe complications such as cholangitis, pancreatitis, cirrhosis, liver failure, or biliary malignancy^1,2^, which makes classification complicated. The signs and symptoms of CM are mostly atypical, the presence of complications is not uncommon, and without early intervention, the risk of developing malignancy among patients with CM is high. Kaneko and Guo et al. found that there are some genetic abnormalities in CM^3,4^. However, early diagnosis in children is challenging, although there are reports in the literature showing that an excellent outcome is possible through timely surgical operations subsequent to a finding of CM during prenatal ultrasonography^5^. Reports regarding prenatally diagnosed CM are relatively scarce, and operative times and surgical outcomes and prognosis are variable^6^. To investigate the clinical significance of early diagnosis, treatment, and intervention, we reviewed CM cases retrospectively. We summarized and compared the general condition, clinical presentation, pathological features, and surgical outcomes in 2 groups of CM patients whose diagnosis was made either prenatally (N = 16) or postnatally (N = 17).

## Materials and methods

### Clinical characteristics

We reviewed and analyzed 33 children who were admitted to the Children’s Hospital of Soochow University between 1 March 2010 and 31 May 2019, and their diagnosis of CM was confirmed by radiological, surgical, and pathological observations. Children’s ages ranged from 0 to 36 months. Patients were divided into a prenatally diagnosed group (N = 16; median age, 4.5 months) and a postnatally diagnosed group (N = 17; median age, 20 months). The radiological techniques used before the operation included ultrasonography, magnetic resonance imaging (MRI), and magnetic resonance cholangiopancreatography (MRCP) for both groups. The clinical presentation, liver function, pathological features, surgical intervention, and clinical outcome were also compared between the 2 groups.

### Statistical analysis

All analyses were conducted using the SAS software V.9.2 (SAS Institute, USA). Normally distributed numerical data are presented as the means ± SD, and categorical data are presented using frequencies and percentages. We analyzed the data using chi-square tests or Fisher's combined probability tests. A p-value less than 0.05 was considered significant.

### Ethics approval and consent to participate

The study protocol was approved by the Institutional Review Board of the Children’s Hospital of Soochow University (No. 20160606013). Written informed consent was provided by the parents or legal guardians of the subjects. All the experimental protocols involving human data were conducted in accordance with national/international/institutional guidelines or the Declaration of Helsinki in the manuscript.

### Consent to publication

All authors have read and approved the content and agree to submit the manuscript for consideration for publication in this journal.

## Results

### General information

**Figure 1:**
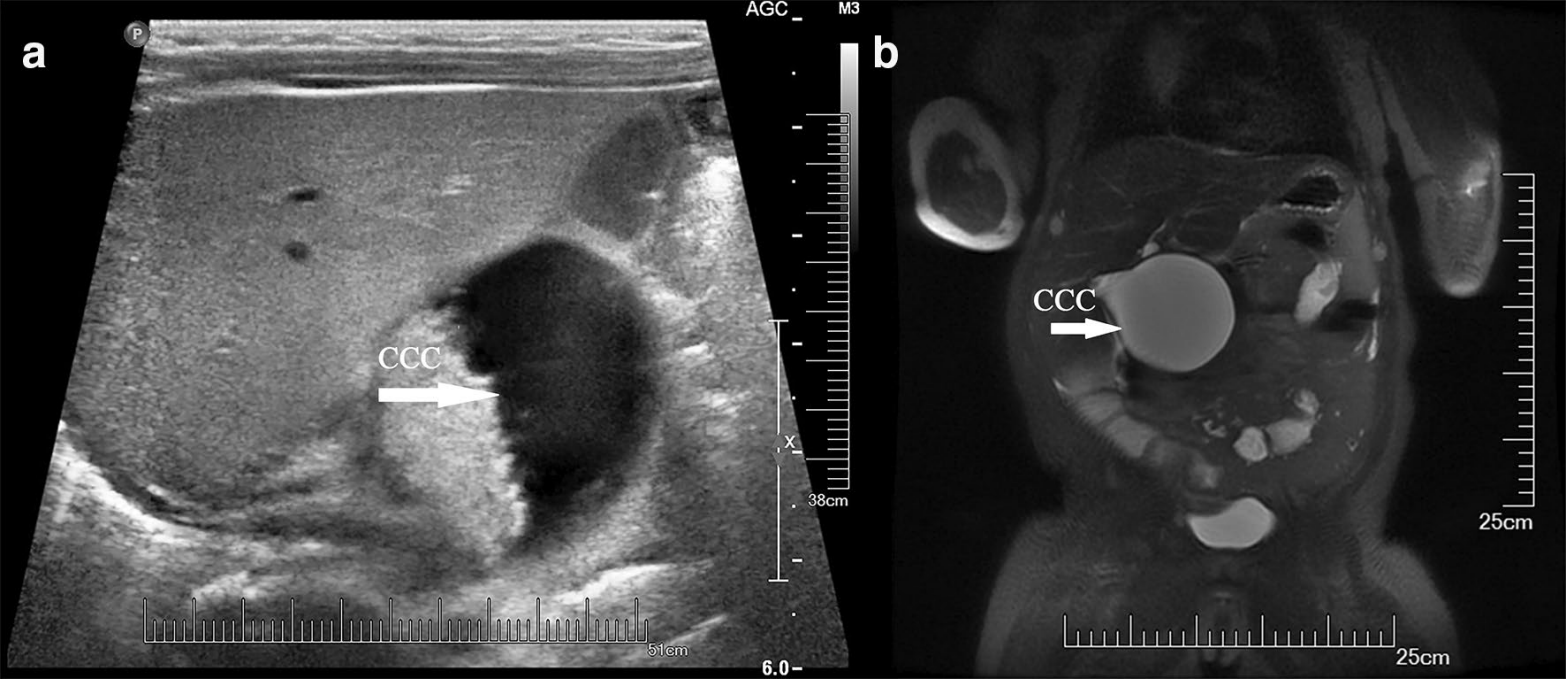
The common bile duct showed cystic dilatation (CCC) on US (**a**) and MRI (**b**) in a prepartum diagnosed CM patient after birth.

A total of 33 CM patients were included in the present study: the prenatally diagnosed group included 15 females and 1 male, whereas the postnatally diagnosed group had 13 females and 4 males. Women were more common in both groups, and the gender difference was not significant between the 2 groups (p > 0.05). The difference in age at operation was significant between the 2 groups (p < 0.05). Table 1 shows the general information about age at operation, anatomy, and liver histology and a longer follow-up for CM. Twenty cases showed common bile duct cystic dilatation, and 13 showed fusiform dilatation (Fig. 1).

**Table 1:** Information on age at operative, anatomy, and the liver histology and a longer follow-up of CM.

	Prenatally (n = 16)	Postnatally (n = 17)
Median age at operation (range)	4.5 m (2–20 m)	20 m (5–36 m)
**Type of common bile duct, n (%)**		
Fusiform	4	9
Cystic	12	8
**Todani’s classification of CBD, n (%)**		
I	9	10
IV	7	7
**PBM type**		
P–C type	5	4
C-P type	8	9
Complex	–	1
Long common channel(No display)	3	3
**Follow up (12–36 months)**		
Early Complication	–	–
Late complication	1 (intestinal obstruction)	–
Liver function normalization(month)	3	6
**Liver histology**		
Fibrosis	1	3

### Clinical features

**Figure 2:**
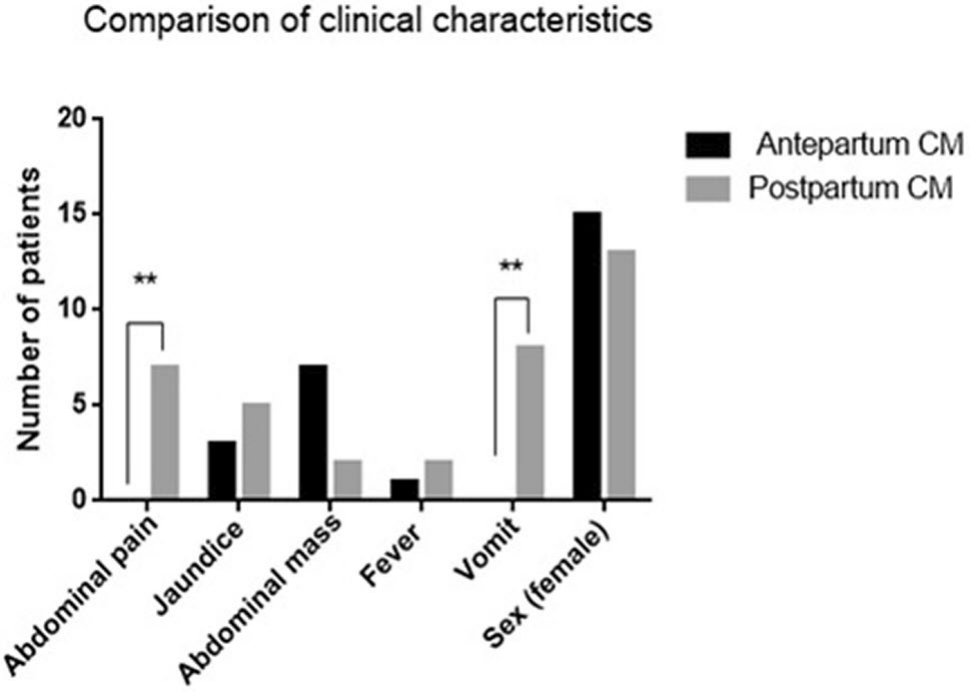
Comparison of clinical characteristics between prepartum-diagnosed CM and postpartum-diagnosed CM.

The triad of CM included abdominal pain, jaundice, and an abdominal mass, with some patients also exhibiting fever and vomiting. In the prenatally diagnosed group, we had 3 patients with jaundice, 7 patients with abdominal masses, and 1 patient with a fever. No patients showed vomiting. Six patients were symptomatic, 2 had acholic stools, and 10 were asymptomatic at birth in the prenatally diagnosed group. In the postnatally diagnosed group, there were 5 patients with jaundice, 7 patients with abdominal pain, 2 patients with abdominal masses, 2 with fever, and 8 patients who exhibited vomiting. The incidences of abdominal pain and vomiting differed significantly between the 2 groups (p < 0.05, Fig. 2).

### Liver function measurements

**Figure 3:**
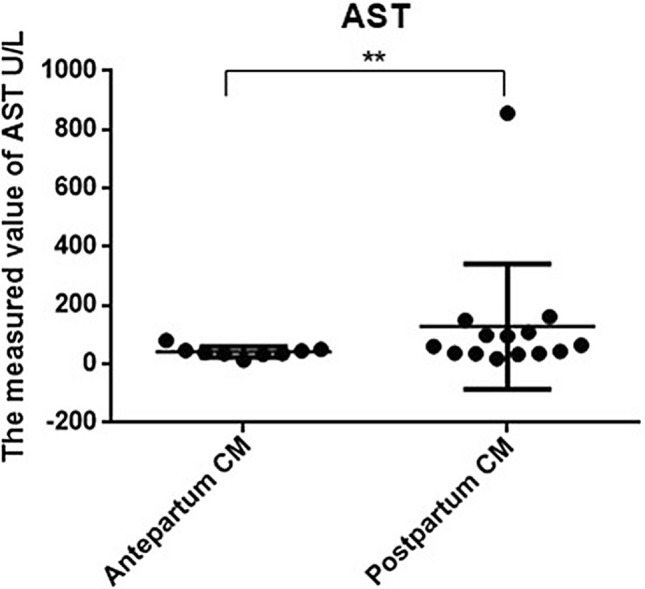
Comparison of liver function using AST between prepartum-diagnosed CM and postpartum-diagnosed CM.

**Figure 4:**
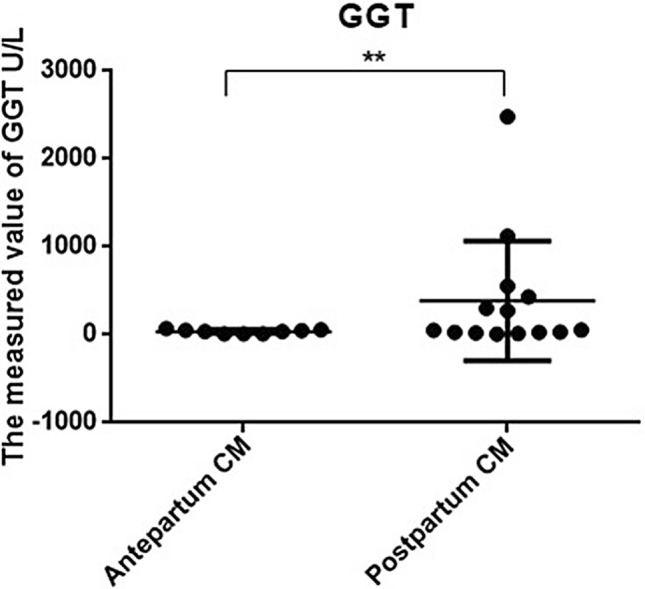
Comparison of liver function using GGT between prepartum-diagnosed CM and postpartum-diagnosed CM.

**Figure 5:**
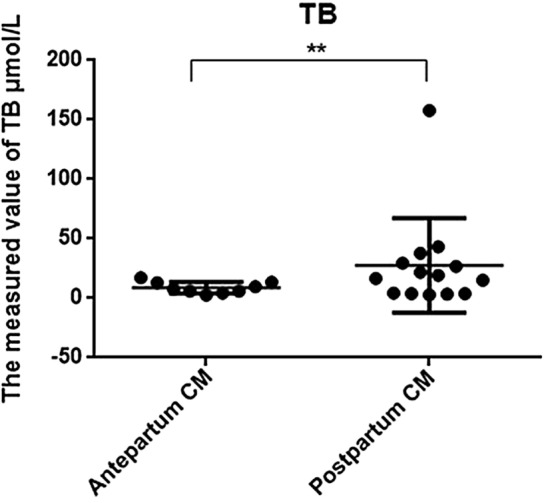
Comparison of liver function using TB between prepartum-diagnosed CM and postpartum-diagnosed CM.

Regarding liver function measurements, AST, GGT, and TB levels in the prenatally diagnosed group were 42.356 ± 17.786 (mean ± SD) U/L, 38.422 ± 21.949 U/L, and 8.8767 ± 4.8367 U/L, respectively, which differed significantly from the same indices in the postnatally diagnosed group (128.61 ± 214.24 U/L, 386.21 ± 679.76 U/L, and 27.606 ± 39.786 U/L, respectively) (p < 0.05). Liver function abnormalities were more common in the postnatally diagnosed group (Figs. 3, 4, and 5).

### Pathologic features

**Figure 6:**
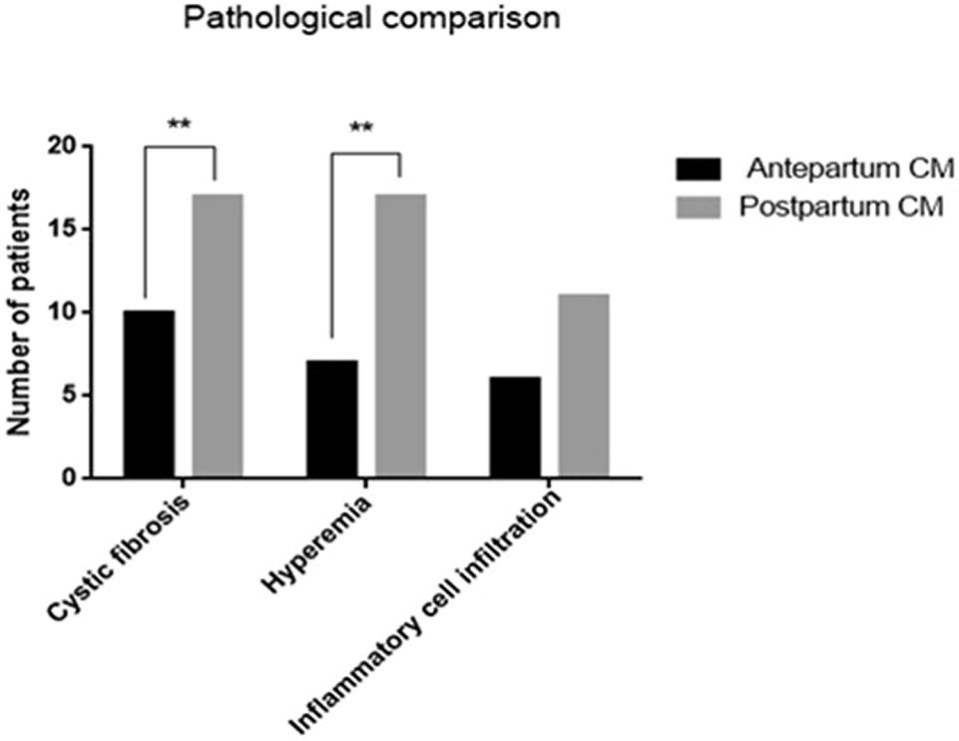
Comparison of biliary tract pathological changes between prepartum-diagnosed CM and postpartum-diagnosed CM.

**Figure 7:**
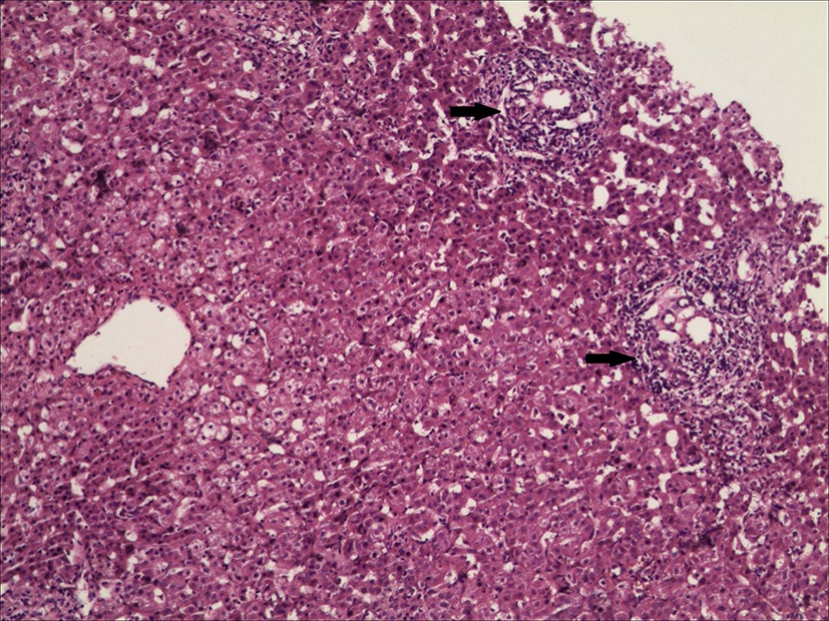
H&E staining showed proliferation of fibrous tissue and lymphocyte infiltration (arrow) in the portal area (× 200 magnification).

Regarding pathological features, after surgery, we collected cyst and gallbladder tissues, stained them with H&E, and observed the tissues microscopically. Both groups showed evidence of inflammation in the cysts and gallbladder. Specifically, in the prenatally diagnosed group, congestion in the cyst wall was observed in 7 patients, inflammatory cell infiltration was observed in 6 patients, and fibrous tissue hyperplasia in the cyst wall was observed in 10 patients. In the postnatally diagnosed group, congestion in the cyst wall was observed in 17 patients, inflammatory cell infiltration was observed in 17 patients, and fibrous tissue hyperplasia in the cyst wall was observed in 11 patients. Congestion and fibrous tissue hyperplasia in the cyst wall differed significantly between the two groups (p < 0.05) (Fig. 6). Four patients underwent liver biopsy and showed hepatic fibrosis (Fig. 7).

### Treatment outcomes

**Figure 8:**
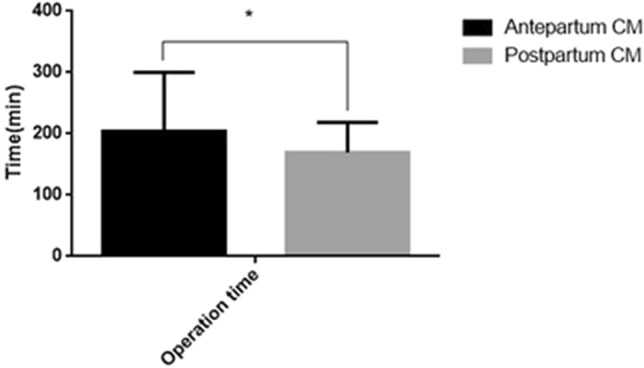
Comparison of operation time between prepartum-diagnosed CM and postpartum-diagnosed CM.

**Figure 9:**
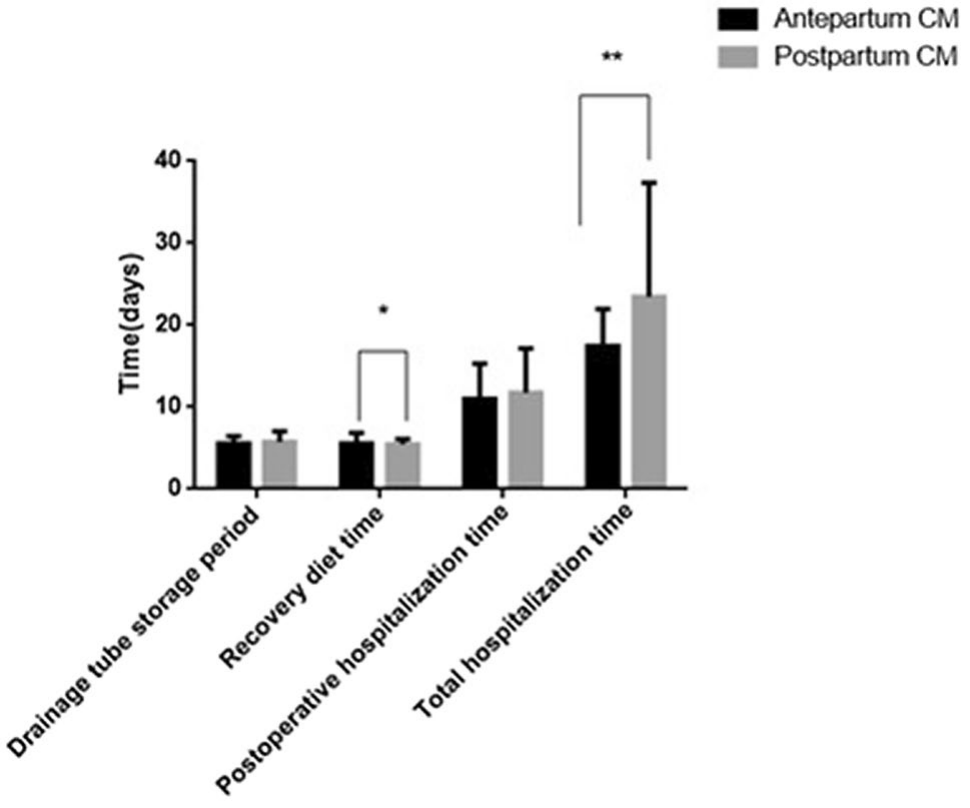
Comparison of the drainage tube storage period, recovery diet time, postoperative hospitalization time and total hospitalization time after the operation between prepartum-diagnosed CM and postpartum-diagnosed CM.

Both groups of patients underwent surgical reconstruction, with operative times of 204.6 ± 95.9 min and 169.7 ± 48.9 min for the prenatally and postnatally diagnosed groups, respectively. Eighteen of these cases had surgery performed using a laparoscopic technique (9 vs 9 in the prenatally diagnosed group and postnatally diagnosed group, respectively), whereas the other 15 cases had surgery performed using conventional open surgery. The time to drainage removal was 5.6 ± 0.9 days and 5.8 ± 1.3 days for the prenatal and postnatal groups, respectively. The time to resumption of a normal diet after surgery was 5.6 ± 1.3 days and 5.5 ± 0.7 days for the prenatally and postnatally diagnosed groups, respectively. The number of hospitalization days after surgery was 11 ± 4.3 days and 11.8 ± 5.4, whereas the total length of hospitalization was 17.4 ± 4.5 and 23.5 ± 13.9 days for the prenatal and postnatal groups, respectively. Operation times, time to resumption of normal diet, and the total length of hospitalization differed significantly between the 2 groups (p < 0.05) (Figs. 8 and 9). The cases were followed up for 12–36 months after the operation. One case in the prenatally diagnosed group had late complications (intestinal obstruction).

## Discussion

### Comparison of clinical characteristics, pathological features, and management between CM patients diagnosed prenatally and postnatally

Almost all postnatally diagnosed patients in the current study had clinical symptoms, chronic biliary inflammation, and abnormalities in hepatic function. The clinical presentation was atypical and most often consisted of nonspecific jaundice, abdominal pain, abdominal mass, fever, and vomiting, with abdominal pain and vomiting being the most common symptoms. In contrast, the prenatally diagnosed patients manifested relatively fewer clinical symptoms, with only 7 of 16 patients presenting with an abdominal mass. This phenomenon might be explained by our early prenatal diagnosis followed by early treatment. However, the prenatally diagnosed group still showed histopathological evidence of fibrous tissue hyperplasia and inflammatory cell infiltration in the cyst wall, which could be the result of pancreatic reflux in pancreaticobiliary maljunction (PBM). This finding is also consistent with our previous finding that most CMs are often associated with PBM^7,8^, and a long common channel can often be found in such cases. Our laboratory tests indicated that abnormal liver function, evident from significantly higher AST, GGT, and TB levels, was more common in CM patients diagnosed postnatally. These results suggested that early surgical intervention was necessary to prevent further liver damage caused by bile duct obstruction. In a previous study, investigators reported additional complications of cirrhosis in prenatally diagnosed CM patients. Okada et al. reported that^9^ prenatally diagnosed CM patients had pathological changes in liver fibrosis even though no clinical symptoms were present. Moreover, liver fibrosis progresses continuously, accompanied by an increased risk of jaundice and abnormal liver function. Therefore, early management is of paramount importance.

In the present study, all patients were treated by primary excision of the choledochal cyst followed by hepaticojejunostomy in a Roux-en-Y fashion. The prenatally diagnosed group had a shorter length of hospitalization and better short-term postoperative outcomes. The surgical outcomes were satisfactory overall, with no incidence of complications or death in either group. Upon long-term follow-up, we did not find biliary obstruction, cholangitis, intestinal obstruction, or bile duct malignancy. These results suggested that early surgical treatment was of great significance in terms of prognosis and in reducing complications in CM patients. Primary excision of the choledochal cyst followed by hepaticojejunostomy in a Roux-en-Y fashion has been adopted as the standard procedure due to its high success rate and low incidence of postoperative complications. During surgery, conditions such as intrahepatic bile duct stenosis and protein plugs and stones within the pancreatic duct and common bile duct need to be corrected to reduce the risk of postoperative complications, including cholangitis, gallstones, cholecystitis, pancreatitis, and biliary malignancies. Overall, the incidence of postoperative complications remains much lower in children than in adults^10^.

The current analysis is inconclusive regarding the optimal time window for the treatment of CM patients. Traditionally, prenatally diagnosed CM patients need to be monitored closely once the baby is born. Treatment of CM patients is related to the age of the symptomatic children. As soon as infants manifest clinical symptoms, surgery should be conducted regardless of infant age. For newborns who do not show clinical symptoms, surgery could be performed when they are 3–6 months old to reduce the risk of anastomosis rupture or stenosis because of a narrow bile duct and to reduce the risk of anesthesia-related adverse events^11^. Research has shown that prenatally diagnosed neonates may have liver damage even without apparent clinical symptoms. In a prospective study by Diao et al.^12^, 36 CM neonates were divided into two groups, with the early treatment group undergoing surgery when the babies were under 1 month of age, whereas the late treatment group underwent surgery after 1 month of age. The results showed that the early treatment group exhibited a significantly lower grade of liver fibrosis, less severe biliary duct inflammation, less dilation of the intrahepatic bile duct, and fewer liver function abnormalities than the late treatment group. They recommended that the operation be performed between 1 and 6 months of age or as early as 1 month after birth, regardless of clinical presentation. However, the optimal time window for the treatment of CM patients needs to be further studied.

### Prenatal diagnosis of CM: imaging techniques and features

Ultrasonography is currently the preferred technique for the prenatal diagnosis of CM, and all prenatal diagnoses of CM in our study were made using this technique. According to the literature, the use of prenatal ultrasonography uncovered CM at 15 weeks at the earliest and averaging at 27 weeks of gestation^13^. The prenatal sonographic features of CM included (1) the diameter of the common bile duct being > 3.1 mm, (2) the cyst usually being located in the region between the lower edges of the liver or hilum and well separated from the gallbladder, and (3) the cyst wall being smooth and slightly thickened. The cyst appeared as an anechoic dark area, with attenuated tension and irregular shape. There was no blood flow in the cyst. (4) A characteristic feature was the connection between the cyst and the intrahepatic bile ducts and gallbladder. (5) The size of the cyst also increased during follow-up^14^.

Biliary atresia (BA) also showed cysts in the liver hilum and should be differentiated from CM, with the sonographic presentation including (1) a relatively small cyst with a diameter less than 2.5 cm, whereas the cyst in CM cases is normally larger (> 4 cm), (2) the size of the BA cyst not changing significantly during follow-up, (3) BA patients usually having an abnormal gallbladder or gallbladder not detectable or detectable but without a recognizable lumen and (4) BA cysts usually observed as round, with a clear border, smooth edge, higher tension and without intrahepatic bile duct dilatation^15^.

Fetal MRI is another modality for noninvasive diagnosis of CM. The “flow void” sign on MR images is used to differentiate the umbilical vein, and the MR image reveals an enlarged head of the common bile duct (CBD), depicting tapering of the body and tail of the CBD^16^. However, this depends on the size of the CM. Only dilatation of the intrahepatic bile ducts may help to differentiate CM from BA.

### Limitations

Our study has several limitations. First, the sample size was relatively small, and selection bias may have been a factor. Second, this study was a retrospective single-center study. A prospective, multicenter study is a necessary future endeavor.

## Conclusions

In summary, compared with prenatally diagnosed CM patients, more symptoms, greater severity of symptoms, and more time was needed to recover after surgery in postnatally diagnosed CM patients. Early diagnosis of CM ensures that treatment can be offered during an optimal time window and ensures satisfactory clinical outcomes and normal development.

## Abbreviations

CBD: Common bile duct
MRI: Magnetic resonance imaging
BA: Biliary atresia
PBM: Pancreaticobiliary maljunction
MRCP: Magnetic resonance cholangiopancreatography
CM: Congenital choledochal malformation
